# Supplementary material for: Protective humoral and cellular immune responses to SARS-CoV-2 persist up to 1 year after recovery
Source: Nat Commun. 2021 Aug 17;12:4984. doi: 10.1038/s41467-021-25312-0 (PMC8370972; doi:10.1038/s41467-021-25312-0)
Supplement: Supplementary file 1 — Supplementary Information [file 41467_2021_25312_MOESM1_ESM.pdf]

Supplementary Data

Title: Protective humoral and cellular responses against SARS-CoV-2 persist up to 1 year after recovery

Chengqian Feng[1], Jingrong Shi[1], Qinghong Fan[1], Yaping Wang[1], Huang Huang[1], Fengjuan Chen[1], Guofang Tang[1], Youxia Li[1], Pingchao Li[2], Jiaojiao Li[1], Jianping Cui[1], Liliangzi Guo[1,3], Sisi Chen[1,3], Mengling Jiang[1], Liqiang Feng[2], Ling Chen[1,2], Chunliang Lei[1], Changwen Ke[4], Xilong Deng[1], Fengyu Hu[1], Xiaoping Tang[1], Feng Li[1].

1. Guangzhou Eighth People's Hospital, Guangzhou Medical University, Guangzhou 510440, China.

2. Guangzhou Institute of Biomedicine and Health, Chinese Academy of Sciences, Guangzhou 510530, China.

3. Department of Gastroenterology, The First Affiliated Hospital, Jinan University, Guangzhou 510632, China

4. Guangdong Provincial Center for Disease Control and Prevention, Guangzhou 511430, China

Correspondence: Xilong Deng (gz8hdxl@126.com), or Fengyu Hu (gz8hhfy@126.com) or Xiaoping Tang (tangxiaopinggz@163.com) or Feng Li (gz8h_lifeng@126.com)

These authors contributed equally: Chengqian Feng, Jingrong Shi, Qinghong Fan, Yaping Wang, Huang Huang, Fengjuan Chen.

These authors jointly supervised this work: Xilong Deng, Fengyu Hu, Xiaoping Tang, Feng Li

Supplementary Figure list

Supplementary Figure 1. Age and gender distribution of follow-up patients.

Supplementary Figure 2. Antibody concentrations in patients before discharge.

Supplementary Figure 3. Correlation of RBD-IgG and Spike-IgG levels overtime.

Supplementary Figure 4. Serum neutralizing capacity between severe and moderate patients.

Supplementary Figure 5. Correlation of microneutralization and Spike-IgG levels.

Supplementary Figure 6. Correlation analysis of microneutralization and RBD-IgG levels.

Supplementary Figure 7. Spot counts of SARS-CoV-2 specific IFN-γ-secreting PBMCs.

Supp Figure 1

a

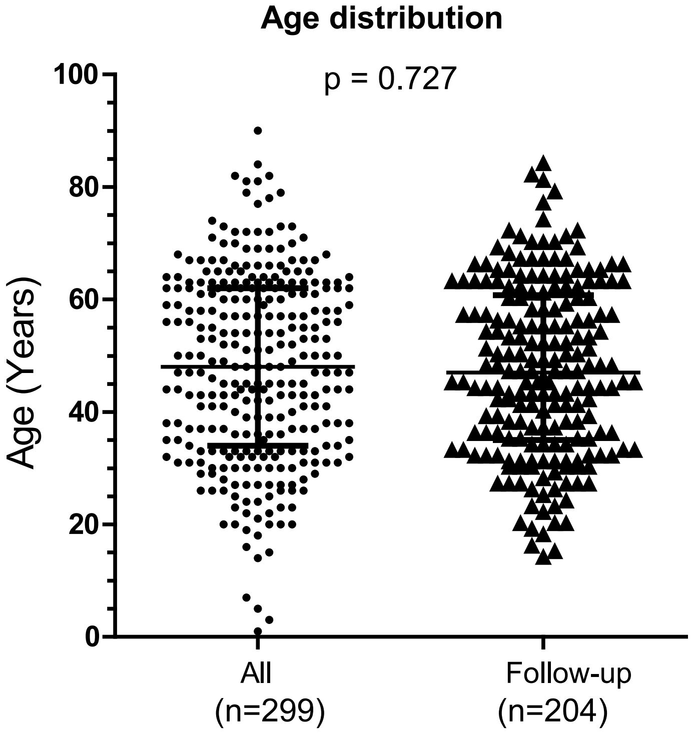

b. Gender distribution of follow-up patients

	Male	Female	Summary	p value
All	140 (46.8%)	159 (53.2%)	299	0.785
Follow-up	93 (45.6%)	111(54.4%)	204	
Summary	233	270	503	

Supplementary Figure 1. Age and gender distribution of follow-up patients. a. Age distribution. All: All patients group, n=299 patients. Follow-up: Follow-up group, n=204 patient. Median with interquartile ranges was shown. The median and 25% to 75% interquartile ranges of all the patients and the follow-up patients were 48 (34 to 62) and 47 (35 to 60.75) years old, respectively. An unpaired t-test with Welch's correction analysis was performed, and the p-value (two-tailed) was >0.05. **b.** Gender distribution. Same to **a.** Frequency and percentage of males and females were shown in the table. A Chi-square analysis was performed, and the p-value (two-tailed) was >0.05. Source data are provided as a Source Data file.

Supp Figure 2

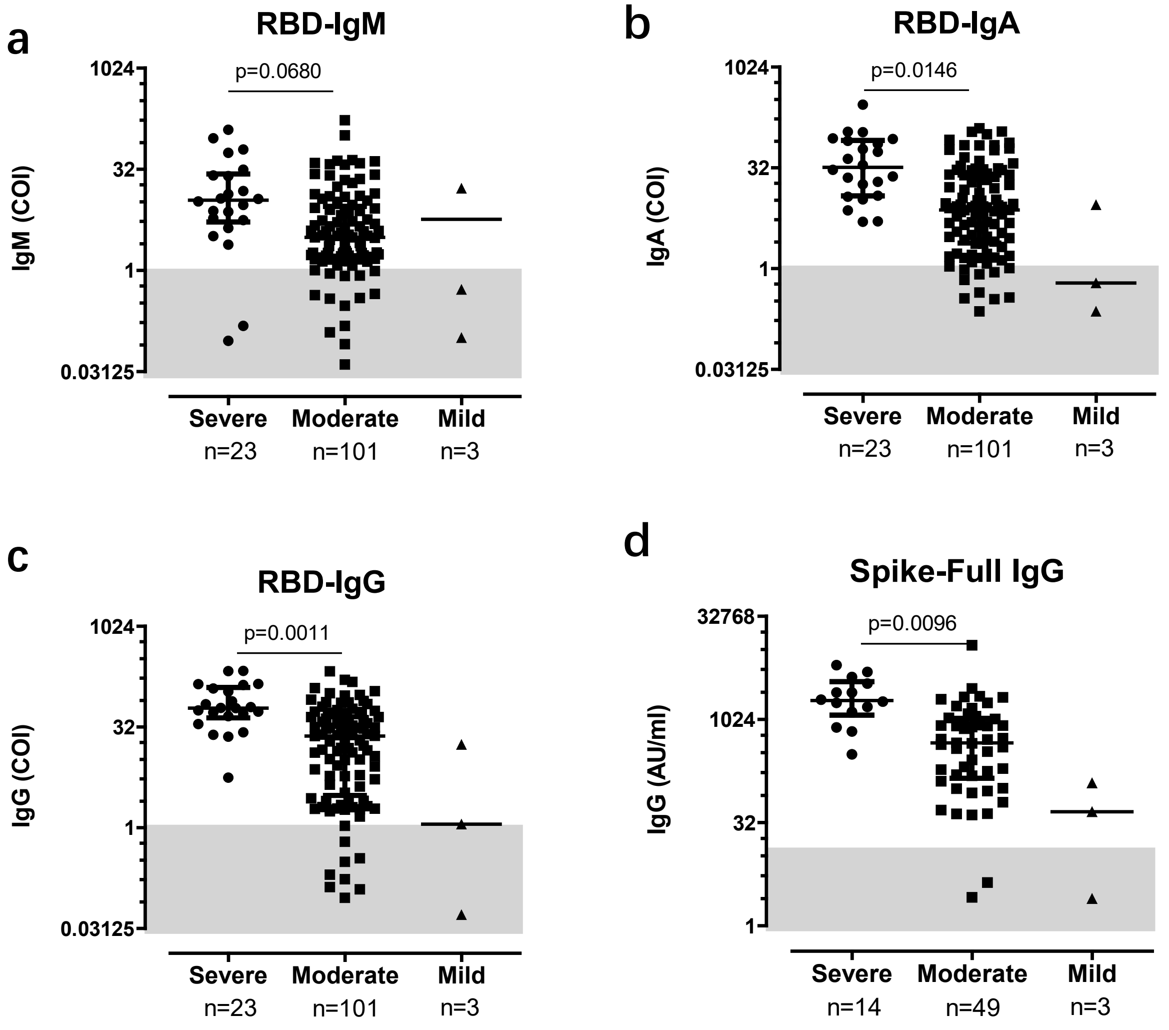

Supplementary Figure 2. Antibody concentrations in patients before discharge. Patients with different clinical symptoms were included in the analysis. The maximal levels of RBD-IgM (**a**), RBD-IgA (**b**), and RBD-IgG (**c**) among multiple time points were chosen to represent the antibody levels of each individual. *x*-axis, severe (n=23), moderate (101), and mild (n=3) group. *y*-axis, antibody levels (COI, log2 scale, cut-off value = 1 COI), detection limit is in shade. d. Levels of full-length Spike binding IgG. *x*-axis, severe (n=14), moderate (49), and mild (n=3) group *y*-axis, antibody levels (AU/ml, log2 scale, cut-off value =10 AU/ml). Data are presented as media with interquartile range. An unpaired t-test with Welch's correction analysis was performed between the severe and moderate groups, but not for the mild group because of the limited sample numbers, and the p-values (two-tailed) were shown. Source data are provided as a Source Data file.

Supp Figure 3

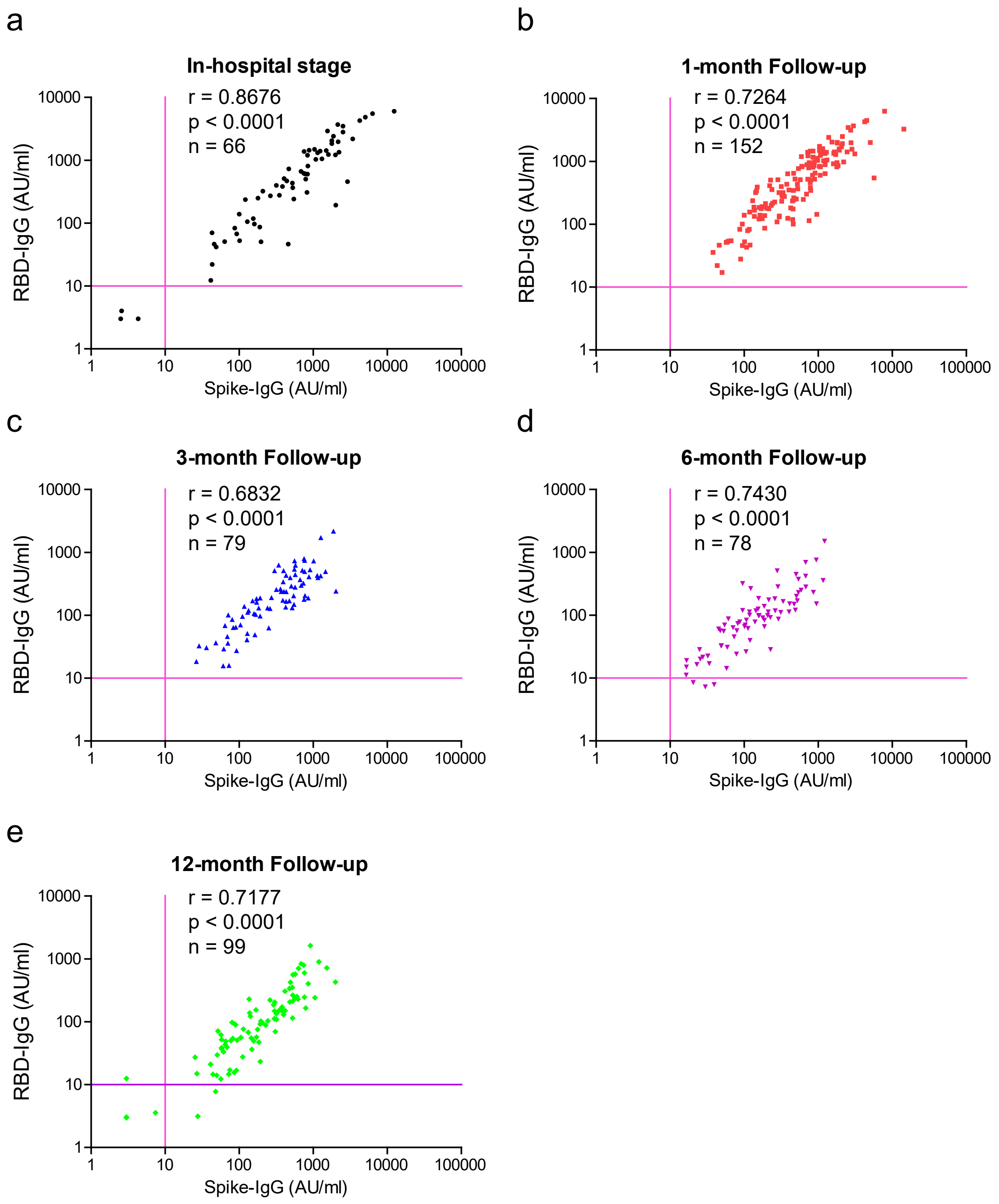

Supplementary Figure 3. Correlation of RBD-IgG and Spike-IgG levels overtime. a. In-hospital stage of COVID-19 patients. **b.** 1-month follow-up stage. **c.** 3-month follow-up stage. **d.** 6-month follow-up stage. **e.** 12-month follow-up stage. *x*-axis, full-length Spike-IgG (Concentration, AU/ml). *y*-axis, RBD-IgG (Concentration, AU/ml). Pearson r numbers and p values (two-tailed) were shown. Source data are provided as a Source Data file.

Supp Figure 4

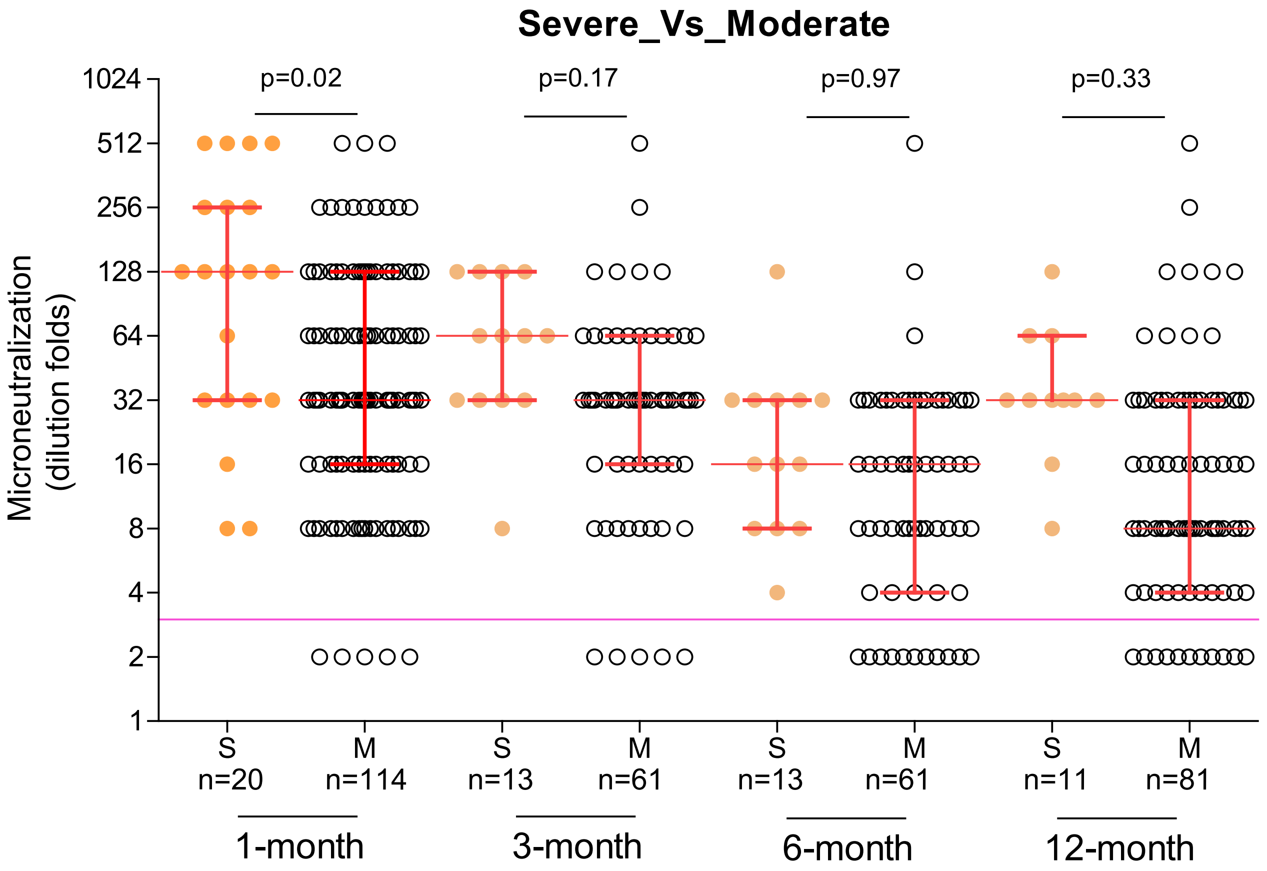

Supplementary Figure 4. Serum neutralizing capacity between severe and moderate patients. See Figure 2a. Only severe (S, yellow dot) and moderate (M, blank circle) patients were included in this analysis. Numbers of individuals at each time point were shown. *y*-axis, microneutralization (dilution folds). The median and 25% to 75% interquartile ranges were shown. An unpaired t-test with Welch's correction analysis was performed, and p values (two-tailed) were shown. Source data are provided as a Source Data file.

Supp Figure 5

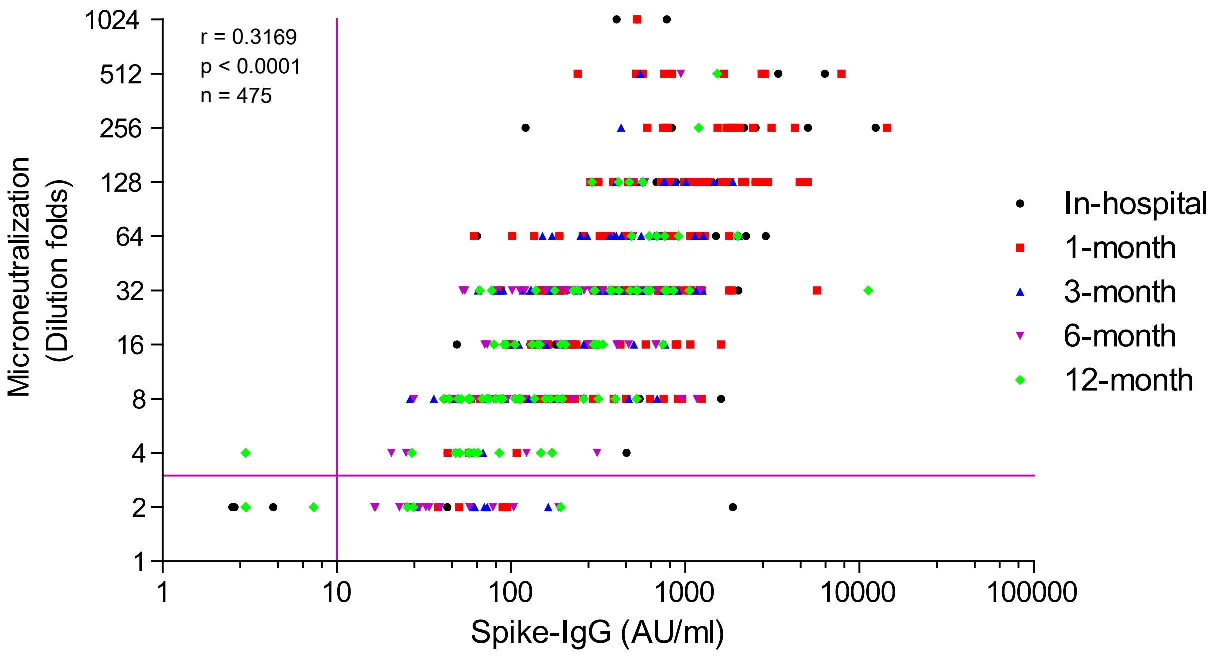

Supplementary Figure 5. Correlation of microneutralization and Spike-IgG levels. *x*-axis, full-length Spike-IgG (Concentration, AU/ml). *y*-axis, Microneutralization (Dilution folds). In-hospital stage (black), 1-month follow-up (red), 3-month follow-up (blue), 6-month follow-up (purple), 12-month follow-up (green). Pearson r number and P value (two-tailed) were shown. Source data are provided as a Source Data file.

Supp Figure 6

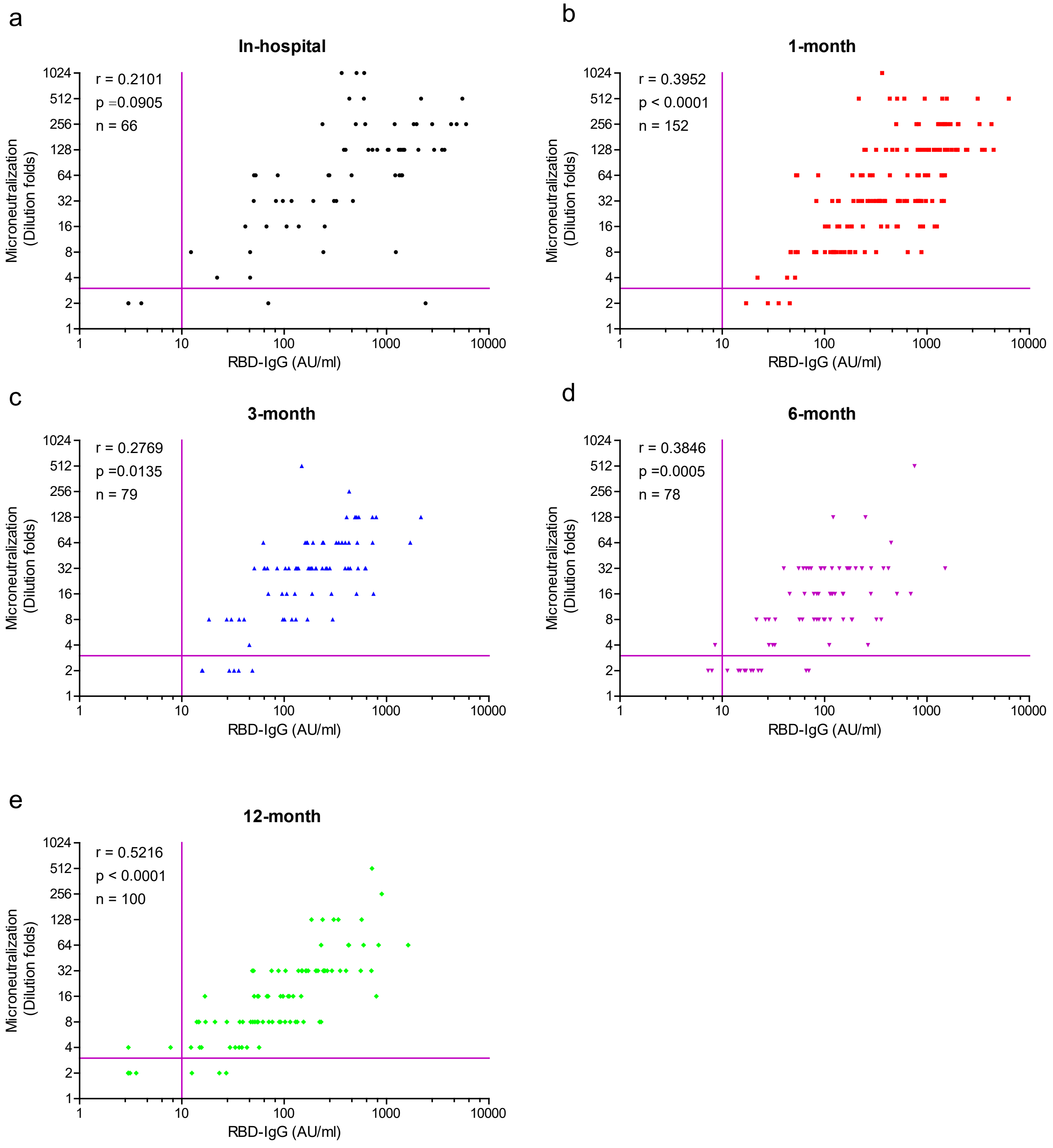

Supplementary Figure 6. Correlation analysis of microneutralization and RBD-IgG levels. Multiple time points post SARS-CoV-2 infection were analysis. **a.** In-hospital stage. **b.** 1-month follow-up. **c.** 3-month follow-up. **d.** 6-month follow-up. **e**. 12-month follow-up. *x*-axis, full-length RBD-IgG (Concentration, AU/ml). *y*-axis, Microneutralization (Dilution folds). Pearson r numbers and p values (two-tailed) were shown. Source Data are provided as a Source Data file.

Supp Figure 7

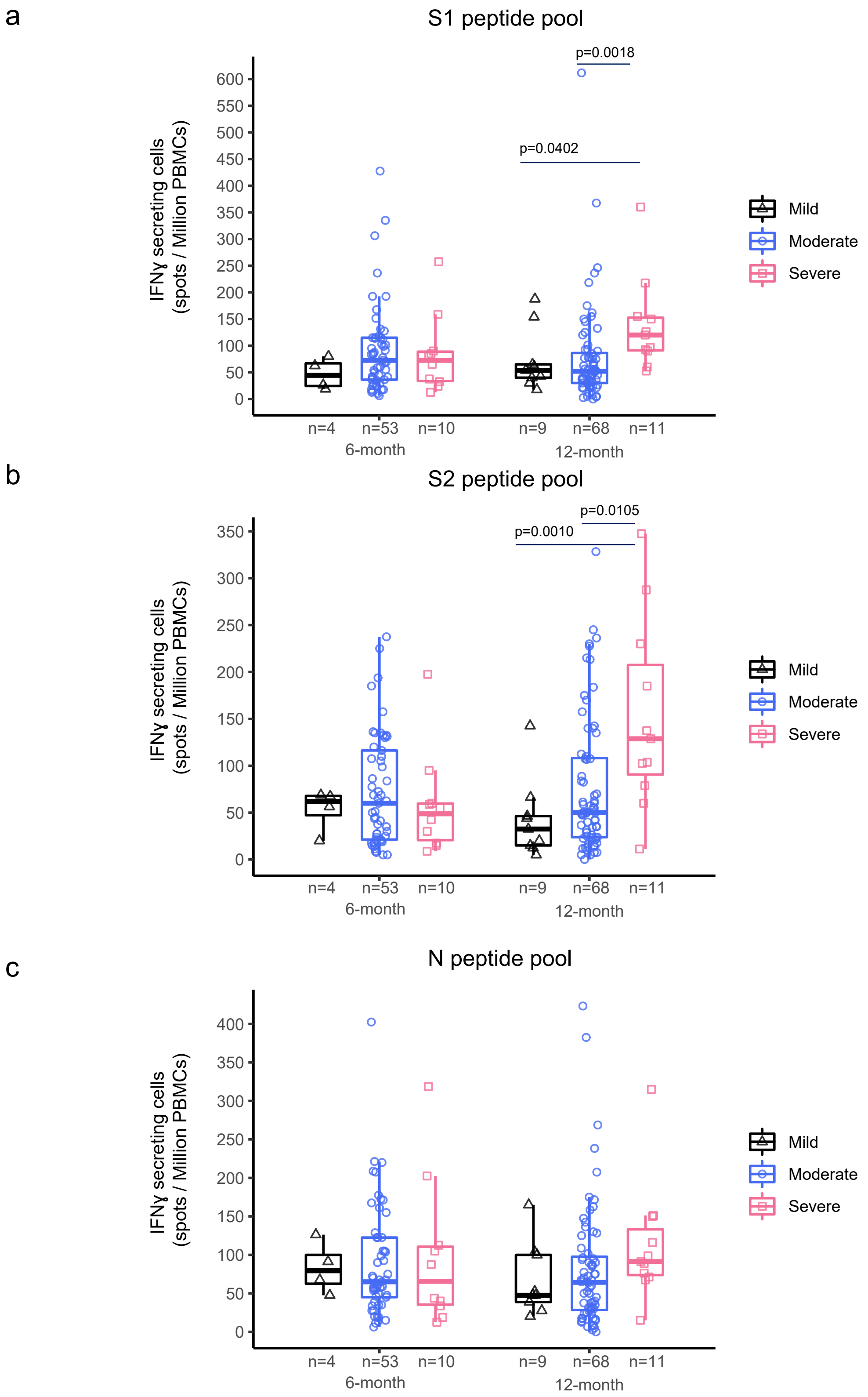

Supplementary Figure 7. Spot counts of SARS-CoV-2 specific IFN-γ-secreting PBMCs. PBMCs isolated from patients at 6-month and 12-month post COVID-19 were stimulated with S1 peptide pool(**a**), S2 peptide pool(**b**), N peptide pool(**c**). Data was represented as box-and-whisker plot (median with interquartile, upperlimit and lowerlimit). Details refer to Fig. 3c. The Mann-Whitney U test was performed. P value (two-tailed) were shown. Source data are provided as a Source Data files.
